# Evaluation of Recurrent Right-Sided Pleural Effusion: Hepatic Hydrothorax vs. Silicone Breast Implant Rupture

**DOI:** 10.7759/cureus.30268

**Published:** 2022-10-13

**Authors:** Mercedes Malone, David Ritchie

**Affiliations:** 1 Internal Medicine, Hospital Corporation of America (HCA) Florida North Florida Hospital, Gainesville, USA; 2 Surgery, Hospital Corporation of America (HCA) Florida Kendall Regional Medical Center, Miami, USA

**Keywords:** video-assisted thoracic surgery, silicone implant rupture, hepatic hydrothorax, talc pleurodesis, pleural effusion

## Abstract

We report a case of a 55-year-old woman who presented to our hospital emergency department with a recurrent right-sided pleural effusion. Her presenting symptom was shortness of breath which first began two years prior after she experienced a blunt thoracic injury. This injury resulted in the rupture of her right silicone breast implant. Since the traumatic rupture of her right breast implant, she developed asthma-like symptoms and allergies that were adequately controlled with bronchodilators, antihistamines, and glucocorticoids. Laboratory investigation was significant for elevated immunoglobulin E (IgE) levels and eosinophilia consistent with an allergic hypersensitivity reaction. She denied a history of smoking, asthma, or allergies preceding the trauma to her right breast implant. Our differential diagnosis also included the possibility of an inflammatory reaction to the silicone breast rupture as a possible etiology for the recurrent pleural effusion.

The patient underwent a right-sided diagnostic and therapeutic thoracentesis procedure on two separate occasions within a span of a month in an effort to improve her symptoms and arrive at a definitive diagnosis. Her worsening symptoms were believed to be triggered by the pleural effusion. Aspirated pleural fluid was sent to the laboratory for analysis. Both samples excluded infectious or malignant causes of the pleural effusion. Ultimately, the source of her pleural effusion was determined to be decompensated liver cirrhosis. The patient underwent a pleurodesis procedure in an effort to seal the pleural space.

## Introduction

Pleural effusions are a common finding among many patients presenting to the emergency department. The underlying etiology for the formation of a pleural effusion includes a wide range of pathophysiologic processes, including congestive heart failure, pulmonary embolism, hepatic, and malignancy [1]. With such a wide range of etiologies, a careful history, physical exam, and dedicated imaging are imperative. Patients who experience recurrent pleural effusions have significantly increased morbidity and increased costs from hospitalizations. 

Pleural effusions are subdivided into bilateral and unilateral presentations. As with bilateral pleural effusions, unilateral pleural effusions have a variety of causes. One condition whereby the findings include a unilateral right-sided pleural effusion is hepatic hydrothorax. This condition occurs when there is a build-up of greater than 500 milliliters of ascitic fluid in the pleural space. The mechanism responsible for hepatic hydrothorax is the movement of ascitic fluid from the peritoneal cavity through a diaphragmatic defect and into the pleural space [2]. One study concluded that the prevalence of hepatic hydrothorax was approximately between 5% to 15% [3]. 

Accurately diagnosing a patient experiencing pleural effusion from hepatic hydrothorax is oftentimes difficult and requires the exclusion of other causes, especially cardiopulmonary. Patients presenting with a right-sided pleural effusion and hepatic hydrothorax must be considered in the differential diagnosis given the position and proximity of the liver. The patient we present not only had cirrhosis but also had a history of a ruptured silicone breast implant. Both conditions can lead to the formation of recurrent right-sided pleural effusion. Due to our patient's past trauma history, we also considered the possibility of a foreign body reaction from a silicone breast implant rupture. While both conditions serve as possible etiologies for recurrent pleural effusion, based upon the analysis of the pleural effusion, clinical data, exclusion of other conditions, and further procedural testing, it was concluded that the recurrent right-sided pleural effusion had arisen from hepatic hydrothorax as opposed to inflammatory changes from the ruptured breast implant.

## Case presentation

A 55-year-old woman presented to our emergency department due to progressively worsening dyspnea and a non-productive cough. Five days before the presentation, she had undergone a thoracentesis at an outside facility whereby one liter of fluid was removed from her right pleural cavity. The outside laboratory pathology was negative for signs of infection or malignancy. Her past medical history was significant for cirrhosis, Roux-en-Y Gastric Bypass (RYGB), asthma, and recurrent episodes of right-sided pneumonia over the past two years.

She reported a history of chronic cough and wheezing after a diagnosis of asthma and adult-onset allergies two years ago. Her asthmatic symptoms reportedly developed after a boating accident two years prior in which she sustained significant trauma to her right thorax. After the incident, she was found to have sustained a ruptured right breast implant. Since her accident, she has not been able to undergo surgery for repair or removal of the implant due to a lack of insurance and sufficient financial resources. Over the past two years, the patient also experienced three separate episodes of pneumonia for which she was prescribed antibiotics which provided only minimal relief for the patient. To note, the patient had never undergone a screening mammogram or colonoscopy.

Upon admission to our facility, the patient was hemodynamically stable and afebrile. Clinical examination was significant for severe diffuse wheezing and right lower lobe crackles upon auscultation of her lungs. Breast examination was remarkable for inversion of the right nipple. Examination of the abdomen revealed a positive fluid wave test consistent with the presence of ascites.

Significant laboratory findings included elevated total bilirubin, mildly elevated AST, and normal ALT. The patient also had elevated serum immunoglobulin E (IgE) of 2000 units/mL and eosinophilia in peripheral blood. These findings were believed to be due to allergic asthma. She denied ever having asthma or allergies until after the trauma to her right breast implant.

**Figure 1:**
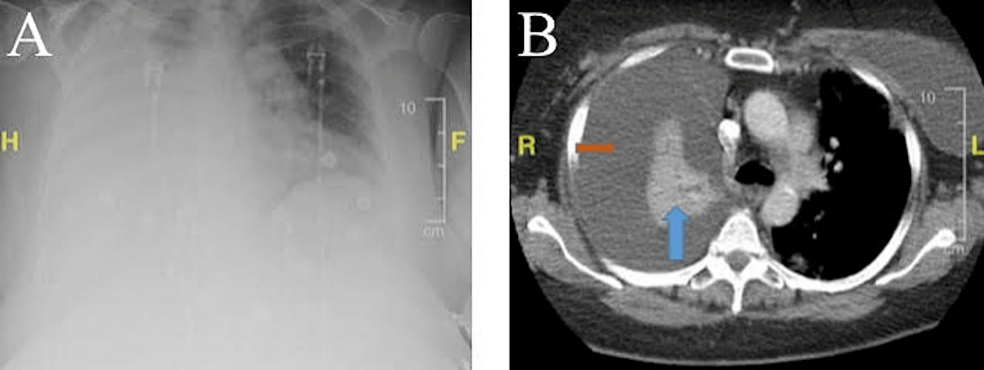
Chest-X Ray and Computerized Tomography (CT) Scan of the Chest Figure 1A: The chest X-ray illustrates a large right-side opacification that is not present on the left aspect of the chest. The right-sided opacity is not specific; it may likely represent a right-sided pleural effusion. Figure 1B: The CT Scan of the Chest illustrates a collapsed right lung, as indicated by the blue arrow. The orange arrow points to the area of the very large pleural effusion that has led to the collapse of the right lung.

**Figure 2:**
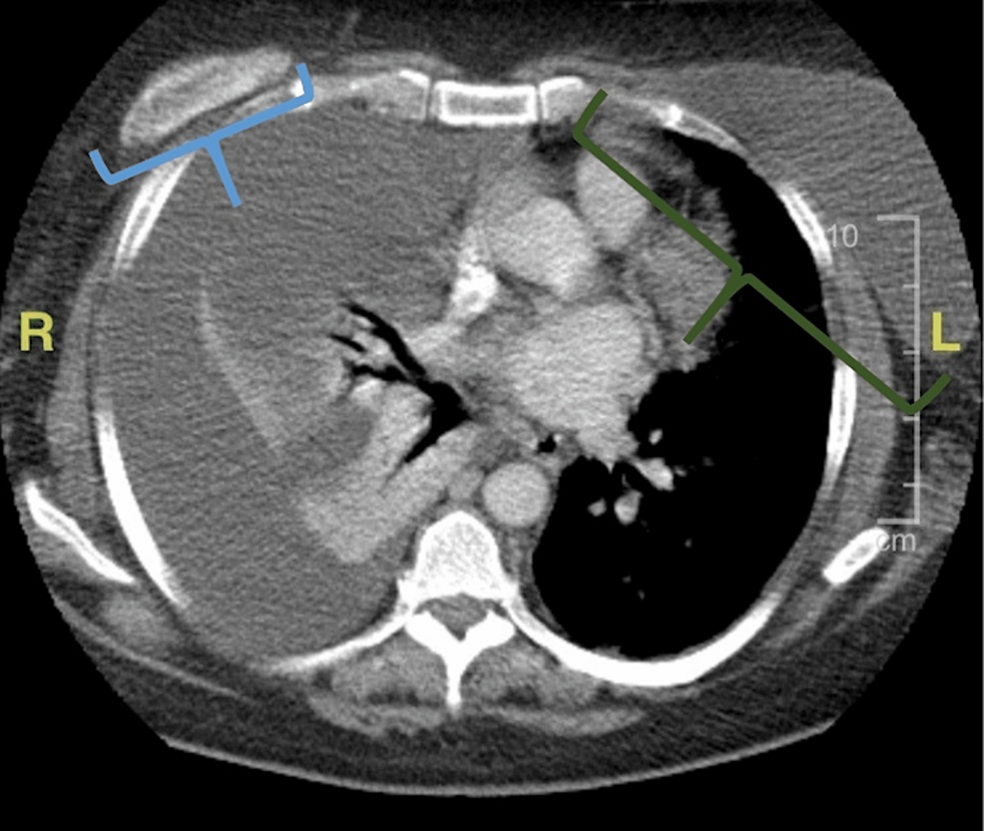
CT Scan of the Chest Computerized tomography scan of the thoracic cavity. The blue bracket on the right encloses the remnant of the silicone breast implant, and the green bracket on the left encloses the intact silicone breast implant. There is a large right pleural effusion causing complete collapse of the right lung and essentially no aeration on the right side.

A chest X-ray was obtained and illustrated nonspecific right-sided opacity (Figure 1A). Based upon a chest CT scan, the presence of a very large pleural effusion was confirmed (Figure 1B). The CT scan of the chest also illustrated the ruptured right breast implant, intact left implant, and complete collapse of the right lung (Figure 2).

**Figure 3:**
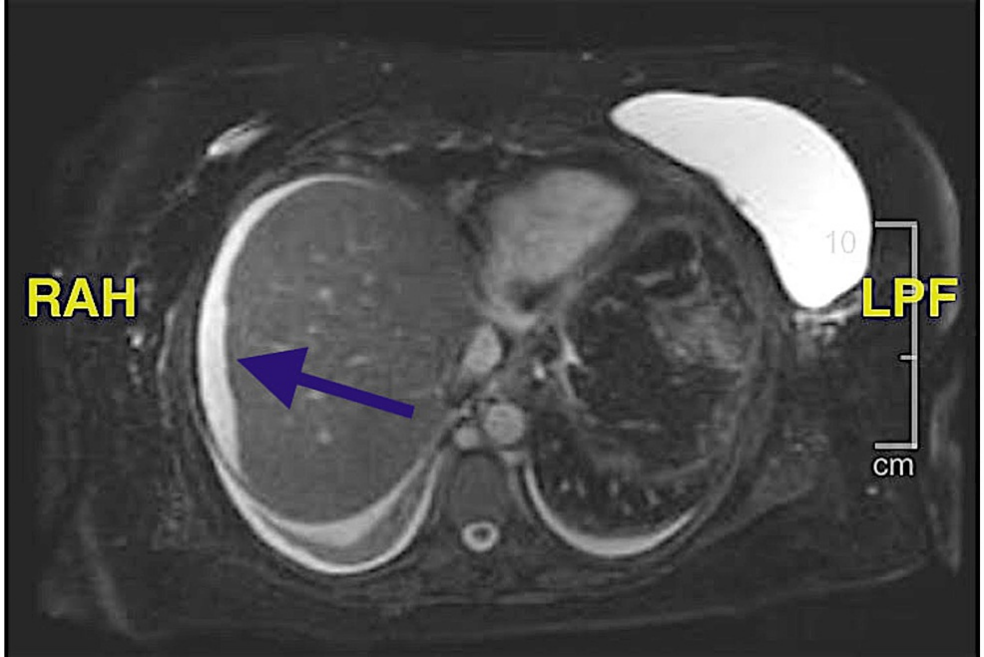
Magnetic resonance cholangiopancreatography (MRCP) The liver appears to be cirrhotic. There is a small amount of ascitic fluid surrounding the right lobe of the liver. The irregular contour of the fluid around the liver suggests that the liver is cirrhotic. The area points to the ascitic fluid encompassing the cirrhotic liver.

The patient underwent a transthoracic echocardiogram which did not demonstrate any significant findings which would explain the etiology of the patient's pleural effusion. An abdominal ultrasound was obtained and revealed abdominal ascites and fatty infiltration of the liver with possible gallbladder sludge. Magnetic resonance cholangiopancreatography (MRCP) illustrated a cirrhotic liver and a mildly dilated gallbladder with a very small common duct without evidence of stones (Figure 3). Although our patient did not exhibit clinical signs of cholecystitis, she was found to have biliary dyskinesia. Her hepatitis panel and extensive autoimmune workup were all negative. Her main risk factor for liver cirrhosis was believed to be due to both alcohol and non-alcohol-related liver disease. The patient was obese and additionally engaged in daily consumption of alcoholic beverages and fatty foods.

**Figure 4:**
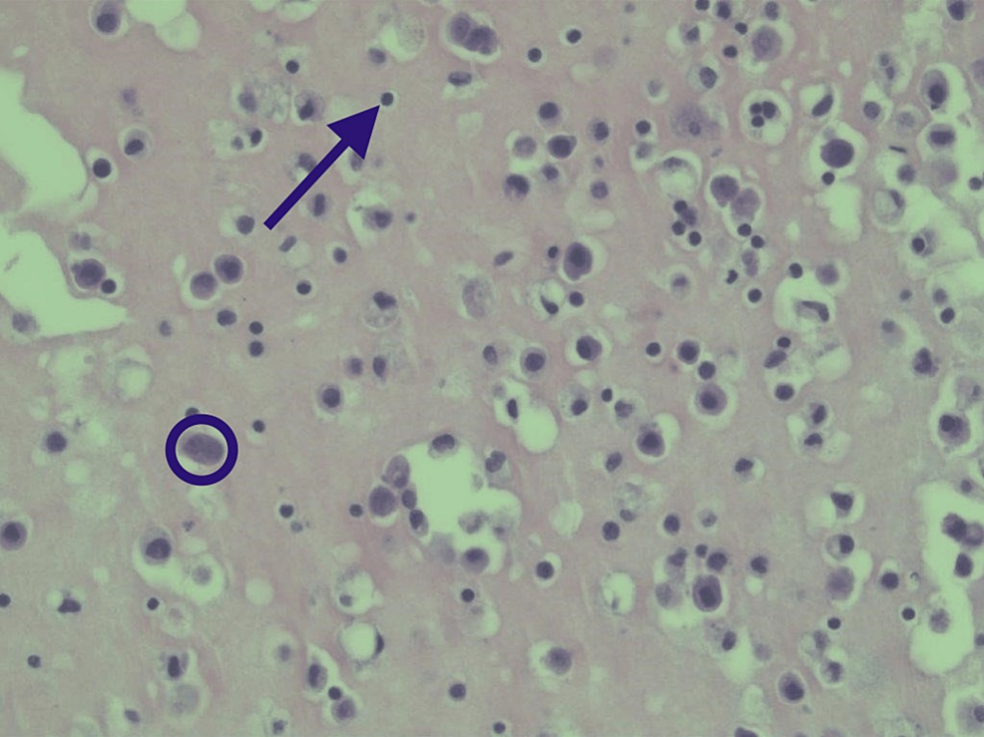
Right Pleural Fluid Cytology No malignant cells are seen. There are large reactive mesothelial cells with a background of chronic inflammation. The circle encloses the larger mesothelial cell, and the arrow points to the smaller lymphocyte. The cytology analysis is normal, without any sign of infection or malignancy. No silicone particles are seen.

The patient underwent a thoracentesis for her large right pleural effusion with a subsequent chest tube placement that had an output of 5 liters within four days. The straw-colored pleural fluid aspirate was sent for analysis (Table 1). Laboratory analysis of the pleural fluid was essentially normal with nonspecific inflammatory cells (Figure 4).

**Table 1:** Right Pleural Effusion Analysis Light’s Criteria was employed in the calculation: With the serum lactate dehydrogenase (LDH) level of 342 units per dl and serum protein level of 6.6 g/dl. The pleural fluid was found to be transudative in nature given that the Pleural Total Protein/Serum Total Protein ratio is less than 0.5 and the pleural lactate dehydrogenase/serum lactate dehydrogenase ratio is less than 0.6. The culture and gram stain were negative for infectious agents.

Fluid pH	7.45
Fluid WBC	148 mm3
Fluid RBC	<1000
Fluid Lymphocytes	51%
Fluid Glucose	109 mg/dl
Fluid Protein	1.2 mg/dl
Fluid Albumin	0.6 mg/dl
Fluid Lactate dehydrogenase (LDH)	58 units/l
Fluid Triglycerides	25 mg/dl

Due to her persistent symptoms and significant chest tube output, the patient underwent Video-Assisted Thoracic Surgery (VATS) with talc pleurodesis. VATS with talc pleurodesis was performed to help reduce the accumulation of fluid in the pleural space and to evaluate for any diaphragmatic defects. During the surgery, there were no adhesions seen, the diaphragm was carefully inspected, and no defects were identified. The effusion was once again aspirated, and then the lung was diffusely aerosolized with sterile talc in the pleural space. The right-sided transudative pleural effusion was resolved upon completion of VATS with talc pleurodesis. Additionally, a bronchoscopy was performed that did not identify any injuries or abnormalities. Upon discharge, it was recommended that she undergo outpatient treatment for her cirrhosis, refrain from the consumption of alcohol, and avoid non-steroidal anti-inflammatory drugs in the setting of talc pleurodesis.

## Discussion

Breast implant rupture is a common complication of breast augmentation, especially in the setting of trauma. The patient had cosmetically augmented the size of her breasts with silicone gel breast implants. After the blunt thoracic trauma to her thorax, her right breast implant had ruptured, and this was evident in the radiological imaging that was performed at our facility. The complications of a ruptured breast implant are variable. The present patient reported the onset of asthma and allergic symptoms after the rupture of her breast implant. Her laboratory workup demonstrated an IgE level of 2000 units/mL consistent with a type I hypersensitivity allergic reaction.

The assessment of a case of a patient with a history of ruptured silicone implant with a cirrhotic liver and subsequent development of recurrent right-sided pleural effusions presented a variety of diagnostic considerations. The main challenge, in this case, was distinguishing the etiology of the pleural effusion. Did the pleural effusion arise as a result of foreign body reaction from the ruptured silicone breast implant, or was it due to hepatic hydrothorax, given her cirrhotic liver?

Silicone breast ruptures have rarely been associated with the formation of pleural effusions. Two previously published reports have noted the occurrence of silicone rupture leading to a foreign body reaction with an ultimate formation of pleural effusion [4,5]. In the case of our patient, the pleural effusion was transudative in nature, with rapid recurrence of the pleural effusion after thoracentesis, and the size of the effusion in the setting of cirrhosis as seen on MRCP, the more probable diagnosis is hepatic hydrothorax. If the etiology was an inflammatory reaction from a rupture silicone breast implant, the effusion would more likely be exudative in nature due to the leak of proteinaceous material from compromised vasculature.

The cytological analysis of the pleural fluid revealed a small number of mesothelial cells which hints at the slight possibility of foreign body reaction to the silicone implant material. However, there were no signs of granuloma formation from the silicone implant material to further substantiate a type IV hypersensitivity reaction. Usually, one would expect to find some silicone particles in the histopathology of the specimen; however, this was not found. While the elevated IgE level suggests the possibility of a type I hypersensitivity allergic response, it is not specific enough to suggest the possibility that the silicone implant was the trigger, as other environmental factors that could be at play.

Based on the pleural effusion's normal pH, negative gram stain and culture studies of the pleural effusion, infectious causes were deemed unlikely. Additionally, the transthoracic echocardiography ruled out congestive heart failure as an etiology. The cytological specimen also lacked any evidence of malignant cells. Exclusion of common conditions such as malignancy, infection, and cardiopulmonary conditions narrowed the source of her pleural effusion to two possibilities; either an inflammatory pleural reaction to silicone from a ruptured implant or translocation of ascitic fluid from decompensated liver cirrhosis. These findings led us to believe that the right-sided transudative pleural effusion was most likely the result of hepatic hydrothorax and less likely a ruptured breast implant.

Treatment of hepatic hydrothorax is difficult [5]. The initial management of hepatic hydrothorax includes the restriction of sodium intake and or diuretics. More invasive interventions such as repeated thoracentesis, continuous positive airway pressure (CPAP), transjugular or surgical portosystemic shunts, pleurodesis, or diaphragmatic repair through VATS with or without pleurodesis and thoracotomy, are often held in reserve for patients refractory to conservative treatments [6]. These interventions can be managed in a step-up approach, as with our patient beginning with repeated thoracentesis and then progressing to pleurodesis before consideration for any shunts. Pleurodesis can be performed with VATS or chest tube, or a combination of both. It is fairly effective, resulting in the resolution of hepatic hydrothorax in around 50% to 85% [6,7].

Initially, our patient had undergone conservative management followed by repeated thoracentesis that proved ineffective in resolving her symptoms. Ultimately a decision was made to attempt a video-assisted thoracoscopic surgery. This minimally invasive technique can offer the resolution of refractory pleural effusions. The procedure involves placing the patient in the appropriate lateral decubitus position and then proceeding with entry into the thoracic cavity with ports that vary in size from 5 mm to 10 mm based on the procedure being undertaken. A double-lumen endotracheal tube or bronchial blocker is utilized in order to implement single-lung ventilation so that the lung on the operative side can be deflated for better visualization [8]. The addition of pleurodesis, as performed in our patient, employs the technique of mechanical (scraping the lung) or chemical (e.g., talc, tetracycline, iodopovidone, etc.) that aids lung adherence to the chest wall [9]. 

## Conclusions

This case highlights the necessity of obtaining a clinical history, focused physical examination, cytopathological analysis of pleural fluid, and VATs surgical procedure to formulate the most probable diagnosis and treatment of recurrent pleural effusion. The underlying source of a pleural effusion is not always clear. Our case demonstrates the importance of a thorough clinical work-up, diagnostic imaging, and laboratory analysis. Recurrent pleural effusions are associated with significantly increased morbidity and hospitalization costs for patients, and it is imperative to diagnose and fully treat the underlying etiology expediently.

## References

[1] Jany B, Welte T (2019). Pleural effusion in adults-etiology, diagnosis, and treatment. Dtsch Arztebl Int.

[2] Cardenas A, Kelleher T, Chopra S (2004). Review article: hepatic hydrothorax. Aliment Pharmacol Ther.

[3] Shaik IH, Gandrapu B, Gonzalez-Ibarra F, Flores D, Matta J, Syed AK (2015). Silicone breast implants: A rare cause of pleural effusion. Case Rep Pulmonol.

[4] von Bierbrauer A, Dilger M, Weissenbach P, Walle J (2008). [Hepatic hydrothorax--a rare cause of pleural effusion that is difficult to manage]. Pneumologie.

[5] Stevens WM, Burdon JG, Niall JF (1987). Pleural effusion after rupture of silicone bag mammary prosthesis. Thorax.

[6] Luh SP, Chen CY (2009). Video-assisted thoracoscopic surgery (VATS) for the treatment of hepatic hydrothorax: report of twelve cases. J Zhejiang Univ Sci B.

[7] Ibi T, Koizumi K, Hirata T, Mikami I, Hisayoshi T, Shimizu K (2008). Diaphragmatic repair of two cases of hepatic hydrothorax using video-assisted thoracoscopic surgery. Gen Thorac Cardiovasc Surg.

[8] Mulholland MW, Albo D, Hawn MT (2015). Operative Techniques in Surgery.

[9] Mierzejewski M, Korczynski P, Krenke R (2019). Chemical pleurodesis - a review of mechanisms involved in pleural space obliteration. Respiratory Research.

